# US gynecologists’ estimates and beliefs regarding ovarian cancer screening’s effectiveness 5 years after release of the PLCO evidence

**DOI:** 10.1038/s41598-018-35585-z

**Published:** 2018-11-21

**Authors:** Odette Wegwarth, Gerd Gigerenzer

**Affiliations:** 1 Max Planck Institute for Human Development, Center for Adaptive Rationality, Lentzeallee 94, 14195 Berlin, Germany; 2 Max Planck Institute for Human Development, Harding Center for Risk Literacy, Lentzeallee 94, 14195 Berlin, Germany

## Abstract

Efficient patient care requires the conscientious use of current best evidence. Such evidence on ovarian cancer screening showed that the screening has no survival benefit but considerable harms; currently no medical organization recommends it. In a cross-sectional online survey study with 401 US outpatient gynecologists we investigated whether they follow the recommendation of their medical organizations in daily practice and report estimates of ovarian cancer screening’s effectiveness that approximate current best evidence (within a ± 10 percent margin of error), and if not, whether a fact box intervention summarizing current best evidence improves judgments. Depending on question, 44.6% to 96.8% reported estimates and beliefs regarding screening’s effectiveness that diverged from evidence, and 57.6% reported regularly recommending the screening. Gynecologists who recommend screening overestimated the benefit and underestimated the harms more frequently. After seeing the fact box, 51.6% revised initial estimates and beliefs, and the proportion of responses approximating best evidence increased on all measures (e.g., mortality reduction: 32.9% [95% CI, 26.5 to 39.7] before intervention, 77.3% [71.0 to 82.8] after intervention). Overall, results highlight the need for intensified training programs on the interpretation of medical evidence. The provision of fact box summaries in medical journals may additionally improve the practice of evidence-based medicine.

## Introduction

The practice of evidence-based medicine (EbM) requires physicians to conscientiously and judiciously use the currently best scientific evidence in making sound decisions about patients’ care. However, beliefs conflicting with scientific evidence have been reported to persist for many years among medical professionals^1,2^, and clinical practices tend to endure despite evidence indicating that they have no benefit for patients or are inferior to other practices^3,4^. Ovarian cancer screening for early detection in average-risk women may be one such inferior practice.

In 2011, the randomized controlled US Prostate, Lung, Colorectal, and Ovarian (PLCO) Cancer Screening Trial^5^—involving 78,216 average-risk women aged 55 to 74 years assigned to undergo either annual screening involving a combination of 6 years of cancer antigen (CA-)125 testing and 4 subsequent years of transvaginal ultrasound (TVU) (screening group) or usual care (nonscreening group)—showed that about 3 women in 1,000 in both the screening and the nonscreening group died of ovarian cancer within that time frame, and about 85 in 1,000 in each group of other causes. It further revealed substantial harms within the screening group: 96 women in every 1,000 screened had a false alarm, of whom 32 had their ovaries unnecessarily removed as part of further diagnostic work-up^5^. As a consequence, the American College of Obstetricians and Gynecologists (ACOG) recommends against screening for ovarian cancer in average-risk women. In 2012, the US Preventive Services Task Force (USPSTF) released a similar recommendation (D recommendation), concluding that there was adequate evidence that annual screening with TVU and CA-125 testing does not reduce ovarian cancer mortality and can lead to important harms, mainly surgical interventions in women without ovarian cancer^6^.

In the present study, set up as a cross-sectional online survey study with two phases (before/after intervention) and conducted five years after the release of the PLCO evidence on ovarian cancer screening’s effectiveness, we asked three questions: First, do US outpatient gynecologists currently recommend ovarian cancer screening? Second, do gynecologists report estimates and beliefs regarding the benefit and harms of ovarian cancer screening by TVU and CA-125 testing that approximate the evidence from the PLCO trial? Third, when provided with an easy-to-understand fact box summarizing the findings from the PLCO trial^5^, do gynecologists revise their initial estimates and beliefs of the benefit and harms of screening if these differed from the evidence?

## Results

### Study Participants

The goal was to survey a national random sample of US gynecologists who practice mainly or exclusively outpatient care because early detection of ovarian cancer is a regular component of their standard clinical practice. To better reflect the general population of US gynecologists, we applied quotas matching the distribution of years in practice and gender of the American Medical Association (AMA) Masterfile at the point of survey completion. 401 gynecologists completed the survey for analysis. The distribution of the demographic characteristics of the final sample matched the distribution of years in practice and gender of the AMA Masterfile (Table 1).

**Table 1:** Distribution of demographic characteristics of the survey sample, compared with the AMA Masterfile for years in practice and gender.

	Sample AMA Masterfile	
	No. (%)	%^a^
Number of participants	401 (100.0)	100.0
Years in practice		
<10	72 (18.0)	18.0
10–19	96 (23.9)	24.0
20–29	96 (23.9)	24.0
≥30	136 (33.9)	34.0
Female	196 (48.9)	49.0
Divided clinical time		
Exclusively outpatient	48 (12.0)	
Mostly outpatient	353 (88.0)	
Practice type		
Gynecologist/Obstetrics	311 (77.6)	
Gynecologist	90 (22.4)	

*Percentages are rounded and may not total 100.

### Gynecologists’ Initial Estimations/Beliefs of the Benefit and Harms of Ovarian Cancer Screening

Of the 401 gynecologists surveyed, 231 (57.6%) reported regularly recommending ovarian cancer screening to average-risk, asymptomatic women. 40.4% estimated—in accordance with current best evidence—the absolute disease-specific mortality reduction due to screening to be zero. 53.9% believed that screening reduces ovarian cancer mortality, with a mean estimate of 21 women per 1,000 screened (range_estimate_: 1 to 994, 95% CI, 10.9 to 31.8). 5.7% thought that more women in the screening group than in the nonscreening group would die from ovarian cancer, with a mean estimated loss of −19.8 women per 1,000 screened (range_estimate_: −1 to −180, 95% CI, −36.4 to −3.2).

55.4% of all gynecologists correctly believed that ovarian cancer screening has potential harms. Overdiagnosis and unnecessary surgical procedures were the harms most frequently named in the subsequent open-end question. Queried on the percentage of false positive diagnoses among all positive diagnoses (96 false positive/101 positive diagnoses per 1,000 screened = 95%, accepted range correct: 86% to 99%), 21.5% of all gynecologists provided estimates that corresponded with evidence. 78.3% underestimated the proportion of false positives, with a mean estimate of 38.6% (range_estimate_: 0% to 85%; 95% CI, 35.6 to 41.6); one gynecologist overestimated it to be 100%. When asked what percentage of falsely alarmed women (96 women per 1,000 screened) would have their ovaries unnecessarily removed (32 women per 1,000 screened) as a consequence of further diagnostic work-up (overtreatment) (32/96 = 33%, accepted range correct: 30% to 37%), 3.2% of gynecologists provided correct estimates. 35.4% underestimated the extent of overtreatment (*M*_estimate_ = 12.0%, 95% CI, 10.5 to 13.4, range_estimate_: 0 to 25), and 61.4% overestimated it (*M*_estimate_ = 73.5%, 95% CI, 71.1 to 75.9, range_estimate_: 40 to 100). Finally, 55.9% of gynecologists correctly thought that the potential benefit of ovarian cancer screening does not outweigh the potential harms.

**Figure 1:**
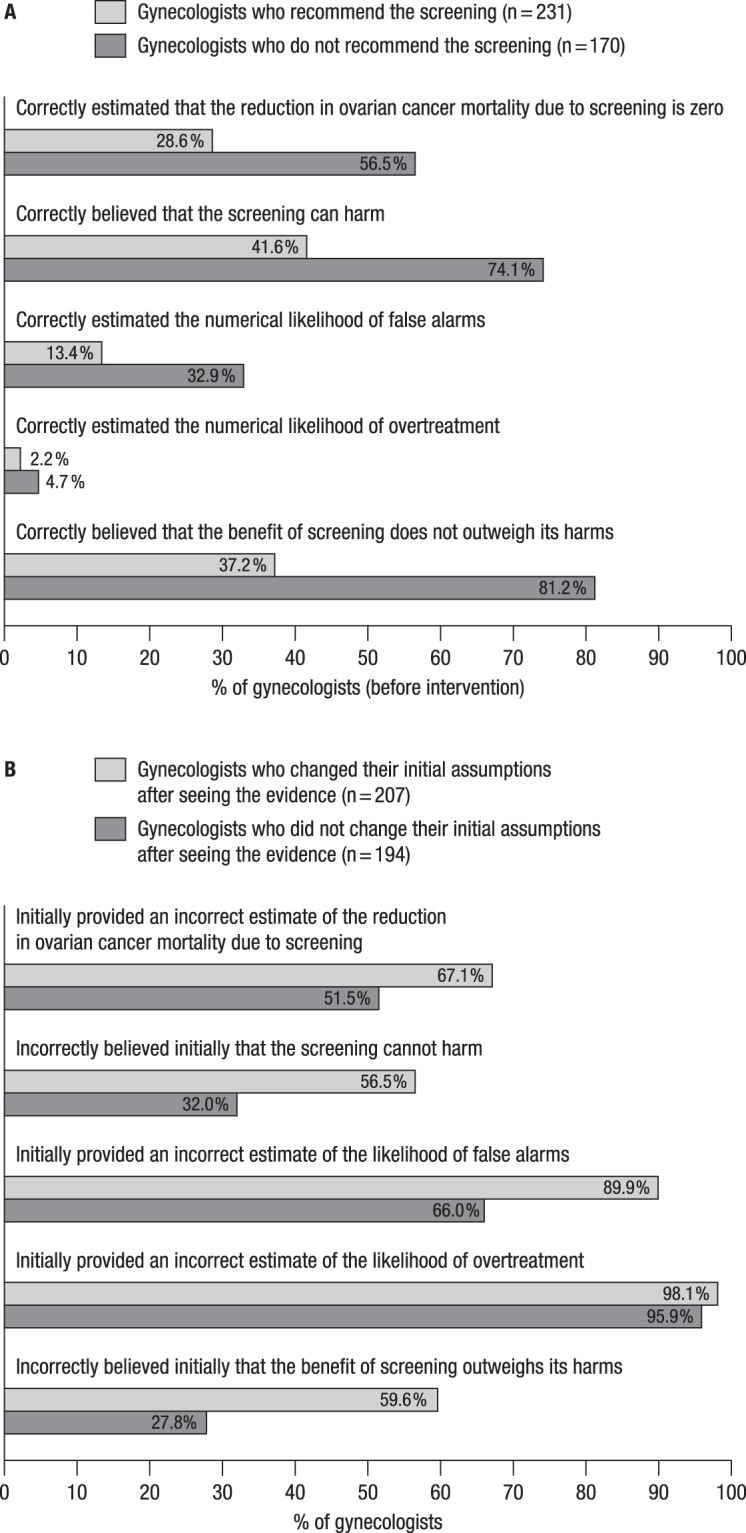
Gynecologists’ initial estimates and beliefs regarding the effectiveness of ovarian cancer screening as a function of their recommendation behavior (**A**) and their initial estimates and beliefs as a function of whether they changed or did not change these after presentation of the PLCO evidence (**B**).

Compared to gynecologists who reported recommending screening (*n* = 231), gynecologists who did not recommend it were nearly twice as likely to provide an estimate of benefit in accordance with current evidence (28.6% [95% CI, 22.8 to 34.9] versus 56.5% [95% CI, 48.7 to 64.0], *p* < 0.001), more frequently believed that the screening has harms (41.6% [95% CI, 35.1 to 48.2] versus 74.1% [95% CI, 66.9 to 80.5]; *p* < 0.001), were less likely to underestimate the likelihood of false alarms (86.6% [95% CI, 81.5 to 90.7] versus 63.5% [95% CI, 55.8 to 70.8]; *p* < 0.001) and of overtreatment (45.0% [95% CI, 38.5 to 51.7] versus 22.4% [95% CI, 16.3 to 29.4]; *p* < 0.001), and were more than twice as likely to view the potential benefit of ovarian cancer screening as not outweighing the potential harms (37.2% [95% CI, 31.0 to 43.8] versus 81.2% [95% CI, 74.5 to 86.8]; *p* < 0.001) (Fig. 1A). We further found that of the gynecologists who recommended screening (*n* = 231), 16.5% (95% CI, 11.9 to 21.9) unexpectedly estimated the mortality reduction to be zero or even negative and simultaneously believed that the screening can cause harms.

### Effect of the Fact Box on Gynecologists’ Estimations/Beliefs of the Benefit and Harms of Ovarian Cancer Screening

**Figure 2:**
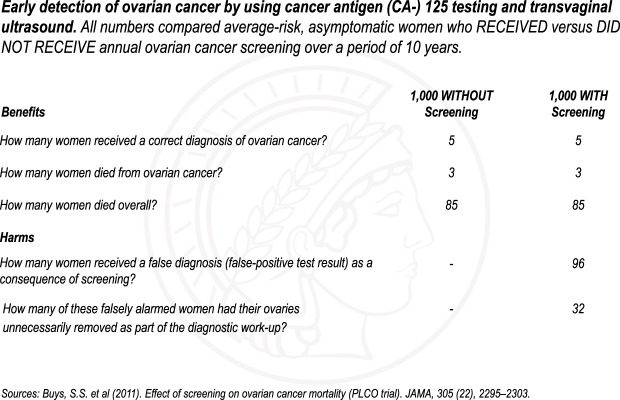
Fact box on ovarian cancer screening summarizing evidence from the PLCO trial.

After responding to the questions, gynecologists were presented with a fact box summarizing the PLCO evidence on the effectiveness of ovarian cancer screening with ultrasound and CA-125 testing^5^ (Fig. 2) and were subsequently asked whether seeing the evidence changed their original estimations.

Of the 401 gynecologists, 194 (48.4%) said that seeing the evidence did not change their estimates; these participants exited the survey without being questioned again. Of these 194 gynecologists, between 27.8% (95% CI, 21.8 to 34.7) and 95.9% (95% CI, 92.2 to 98.2), depending on the question, had initially provided estimates and beliefs that diverged from current best evidence (Fig. 1B).

Of the 207 gynecologists (51.6%) who responded that seeing the evidence changed initial estimates, outcomes improved on all measures (Table 2): The mean estimate for cancer-specific mortality reduction decreased from 14.2 to 0.4 per 1,000 women (range_before_: −180 to 994, *SE*_before_: 5.36; range_after_: −52 to 75, *SE*_after_: 0.64; *p* < 0.001), and the mean estimated percentage of false positives among all positive test results increased from 43.2% (range_before_: 0% to 99%, *SE*_before_: 2.02) to 65.9% (range_after_: 1–100%, *SE*_after_: 1.77) (*p* < 0.001). Similarly, the number of gynecologists who believed the screening to have potential harms increased from 43.5% to 87.0% (*p* < 0.001), and the number who thought the potential benefit of screening outweighed the potential harms decreased from 59.6% to 30.0% (*p* < 0.001).

**Table 2:** Effect of the fact box on the knowledge of the 207 gynecologists who said that seeing the evidence from the PLCO trial changed their estimates.

	Number of physicians providing the correct response (%; 95% CI)	
	Before seeing the evidence	After seeing the evidence
**Knowledge on Benefit**		
Reduction of ovarian cancer mortality?		
- Correct estimate (0 out of 1,000)	68 (32.9; 26.5 to 39.7)	160 (77.3; 71.0 to 82.8)
- Overestimate	128 (61.8; 54.8 to 68.5)	33 (15.9; 11.2 to 21.7)
- Underestimate	11 (5.3; 2.7 to 9.3)	14 (6.8; 3.7 to 11.1)
**Knowledge on Harms**		
Do you think that ovarian cancer screening can also harm a woman?		
- Yes (correct)	90 (43.5; 36.6 to 50.5)	180 (87.0; 81.6 to 91.2)
How many of every 1,000 women attending ovarian cancer screening over a period of 10 years do you think will receive a positive test result? (X out of 1,000 screened)		
- Correct estimate (101 women; ±10%: 91 to 111)	24 (11.6; 7.6 to 16.8)	66 (31.8; 25.6 to 38.7)
- Underestimate (<91)	171 (82.6; 76.7 to 87.5)	138 (66.7; 59.8 to 73.0)
- Overestimate (>111)	12 (5.8; 3.0 to 9.9)	3 (1.5; 0.3 to 4.2)
How many of these positive test results do you think are false-positive test results? (%)		
- Correct estimate (95%; ±10%: 86% to 99%)	21 (10.1; 6.4 to 15.1)	67 (32.4; 26.0 to 39.2)
- Underestimate (<86%)	186 (89.9; 84.9 to 93.6)	139 (67.1; 60.8 to 74.0)
- Overestimate (100%)	—	1 (0.5; 0.0 to 2.7)
How many of these women who received a false-positive test result will have their ovaries removed as a consequence of further diagnostic work-up? (%)		
- Correct estimate (33%; ±10%: 30% to 37%)	4 (1.9; 0.5 to 4.9)	49 (23.7; 18.1 to 30.1)
- Underestimate (<30%)	84 (40.6; 33.8 to 47.6)	47 (22.7; 17.2 to 29.0)
- Overestimate (>37%)	119 (57.5; 50.4 to 64.3)	111 (53.6; 46.6 to 60.6)
Do you think that the potential benefit of ovarian cancer screening (e.g., reduction of disease-specific mortality) outweighs the potential harms (e.g., false positives, overdiagnosis)?		
- No (correct)	84 (40.4; 33.3 to 47.6)	145 (70.0; 63.3 to 76.2)

Gynecologists were more likely to change their initial estimations/beliefs after seeing the evidence if they had been practicing for longer and had initially provided fewer correct estimations/beliefs of screening’s benefit and harms (logistic regression results, see Supplementary Information).

## Discussion

In 2011, evidence from the PLCO trial demonstrated that ovarian cancer screening in average-risk, asymptomatic women resulted in no benefit, but considerable harms. On the basis of this evidence, in 2012 the USPSTF and the ACOG recommended against screening for ovarian cancer in these women. Five years after the release of the evidence and 4 years after these recommendations, we found that ovarian cancer screening persists in clinical practice, with 57.6% of 401 outpatient gynecologists saying that they regularly recommend ovarian cancer screening to average-risk women for early detection. Our study further showed that a majority of gynecologists in our sample assumed the benefit-harm ratio of the screening to be more favorable than indicated by current best evidence: more than half overestimated the benefit, nearly 80% underestimated the proportion of false-positive results, and over 96% under- or overestimated the extent of screening-related overtreatment. Gynecologists who said they recommended screening were less likely to provide estimations of the benefit and harms of the screening that approximated the evidence than were gynecologists who said they did not recommend it.

Why do a considerable number of gynecologists, 5 years after the release of the PLCO evidence on ovarian cancer screening, estimate its benefit and harms in numbers that diverge from current best evidence by a ± 10 percent margin of error and more? One reason might be that some gynecologists do not know how to interpret the health statistics provided by the trial^7,8^. Previous studies found that physicians are misled by framing effects created by relative as opposed to absolute risk formats^9–13^, have difficulty calculating the positive predictive value of tests^14–17^, or have trouble understanding screening statistics^18,19^. Our present study indicates for the first time that a fact box summarizing the scientific evidence in an easy-to-understand frequency format can partially solve this problem in clinicians: after reading the fact box, more than half of the gynecologists in our sample revised and improved their original estimates and beliefs regarding ovarian cancer screening’s effectiveness. Gynecologists who had initially provided fewer correct estimations/beliefs of screening’s effectiveness and who had been practicing for longer were particularly receptive to the presentation of the evidence in the fact box.

Yet statistical illiteracy cannot explain why 37.2% of the 231 gynecologists in our sample who said they recommended screening did not think that the benefit outweighed the harms and why 16.5% of these 231 saw no benefit at all, but only harms. Two potential explanations are the practice of defensive medicine^20,21^ and conflicting interests^22–25^. Also, evidence from randomized trials may conflict with the conventional wisdom that “early caught” means “successfully fought.”

The study has limitations. First, we cannot explain why some gynecologists do not revise their estimations diverging from evidence. However, we can largely rule out the possibility that these physicians either were not aware of the best available evidence or did not know how to interpret it: the fact box summarizing the results of the PLCO trial acquainted them with this information and should have been easy for them to interpret, given that the format has been shown to work effectively even for laypeople with low literacy levels^26,27^. Second, we can only speculate why gynecologists with more years in practice were more receptive to the evidence presented in the fact box than were gynecologists with fewer years in practice. The implementation of evidence-based medicine and its tools in medical training is a fairly new development over the last 10 to 20 years. Some of the gynecologists whose medical education took place prior to that might have felt less confident about how to interpret medical evidence and thus been more open to guidance provided by the fact box. Third, the adequacy and precision of the figures from the PLCO trial as an external criterion of what counts as a good proxy for screening’s effectiveness might be questioned. We addressed this potential concern by setting a ±10 percent margin of error for the reported figures when evaluating gynecologists’ estimates, except for mortality reduction. In the PLCO trial, the reported nonsignificant mortality risk ratio of 1.18 in disfavor of the screening group led its authors to conclude “that the boundary for futility had been reached” (7, p. 2300). Some may nonetheless argue that the reported 95% CI of 0.82 to 1.71 may include the likelihood of a benefit in favor of the screening group. Hypothetically, assuming the lowest boundary of the 95% CI, 0.82, to be the real effect of the screening, about 0.5 women per 1,000 were saved from ovarian cancer death due to screening. If we had thus rated not only “zero out of 1,000 screened” but also “1 out of 1,000 screened” as a good proxy of the mortality reduction due to screening, the observed percentage of correct estimates would have increased only slightly, from 40.4% to 41.6%. One can further argue that using the United Kingdom Collaborative Trial on Ovarian Cancer Screening (UKCTOCS) trial^28^—released around the same time as when we pursued our study—would have been a more appropriate external criterion for judging gynecologists’ assumptions about the screening’s effectiveness. Yet, contrary to what was implied by the press release and the subsequent media coverage, the UKCTOCS trial did neither demonstrate that screening reduces ovarian cancer-specific mortality nor all-cause mortality, but instead confirmed the results of the PLCO trial regarding a non-significant mortality reduction and considerable harms due to the screening^29^. For this reason, the findings of UKCTOCS trial left unchanged all evaluations on the screening’s effectiveness and the subsequent recommendations of major medical organizations (e.g., USPSTF). Only exploratory analyses suggested a potentially delayed, still non-significant mortality benefit after 10 years of follow-up for one screening arm—the multimodal screening arm using CA-125 serum testing interpreted with use of the ‘Risk of Ovarian Cancer Algorithm [ROCA]’—but only when certain subgroups of this screening arm were excluded from analyses^29^. Therefore, medical organizations requested further years of follow-up to see if the trend will be substantiated. Thus, whether assigning women to multimodal screening will eventually be (cost-)effective and a feasible screening tool with a favorable benefit-harm ratio for the future needs still to be proven. Fourth, we cannot rule out the existence of nonrespondents’ bias. Although we achieved a reasonable response rate and stratified the sample to match gynecologist characteristics for years in practice and gender in our sample to the AMA Masterfile at survey completion, we cannot exclude the likelihood that gynecologists who were more attracted to the topic of evidence on ovarian cancer screening were more likely to respond to the survey. If this were the case, however, our results may even underestimate the problem of gynecologists’ estimations diverging from evidence. Fifth, due to the cross-sectional design of the study we do not know if gynecologists retain the information from the fact box over a longer run, nor do we know if and how it impacts their day-to-day recommendation and counseling behavior. Sixth, because the intent of the study was not to test for superiority of fact boxes over alternative information formats (e.g., icon arrays, written text) in informing physicians about medical evidence, our study design did not contain a control arm. Although our results indicate that fact boxes can improve physicians’ knowledge of medical evidence, these do not establish the superiority of fact boxes. Finally, only US gynecologists were included in our study, which may affect the generalizability of the results. However, given that other studies^7,16^ and a recent comprehensive review^2^ documented comparable misunderstandings about the effectiveness of different cancer screenings among physician populations from various countries, we presume that our findings are not restricted to US gynecologists only.

Despite these limitations, our findings suggest that the currently used procedure of ovarian cancer screening may tend to stay in place because a considerable number of gynecologists overestimate the screening’s benefit and underestimate its harms. This may likely prevent them from responding accurately to their patients’ questions about the effectiveness of screening, which in turn hinders patients in making an informed choice. On the positive side, a simple evidence-based fact box may effectively increase physicians’ understanding of current best evidence^30^. Our findings may encourage both editors of medical journals to incorporate fact box summaries in clinical research articles^31^ and medical educators^32^ to implement trainings on how to correctly interpret medical evidence in general and screening statistics in particular in their medical curricula.

## Methods

### Study Oversight

The study was set up as a cross-sectional online survey study with two phases (before/after intervention). Its content and design were developed by the authors, piloted with 5 gynecologists, and revised after feedback. The Institute for Consumer Research (GfK) (Nuremberg, Germany) programmed the online version of the survey and conducted the online survey by using the SERMO physician panel as sample frame. The study was performed in accordance with relevant guidelines and regulations, and informed consent was obtained from all participants prior to the study. The study protocol was approved by the Institutional Review Board (IRB) of the Max Planck Institute for Human Development.

### Sample Frame

The sample frame was the SERMO physician panel maintained by SERMO and subcontracted by the GfK. The SERMO physician panel comprises about 160,000 US physicians across all major medical specialties and about 18,320 physicians with the specialty gynecology/obstetrics. Panelists have agreed in advance to participate in online research. All panel members complete a detailed profiling survey relating to their specialty and applicable subspecialties, years of practice, workplace setting, conditions treated, patient load per condition, and procedures conducted at the time of registration. All panel members need to verify their credentials in an accurate verification process and update the profiling survey on an annual basis.

### Study Procedure and Participants

The goal was to survey a national random sample of US gynecologists who practice mainly or exclusively outpatient care because early detection of ovarian cancer is a regular component of their standard clinical practice. To better reflect the general population of US gynecologists, we applied quotas matching the distribution of years in practice and gender of the American Medical Association (AMA) Masterfile at the point of survey completion. To detect differences of 20% or higher with 90% power in the proportion of gynecologists’ correct estimates and beliefs regarding screening’s effectiveness before and after intervention (2-sided alpha of 0.05), we calculated that a sample size of 400 physicians was needed. To allow for nonresponse and ineligibility upon invitation, the GfK drew a random sample of 980 US gynecologists from SERMO’s physician panel in May 2016 and contacted them by email. The email provided basic information about the study, the link to the survey (with personalized password), and an offer of a $50 honorarium upon survey completion.

**Figure 3:**
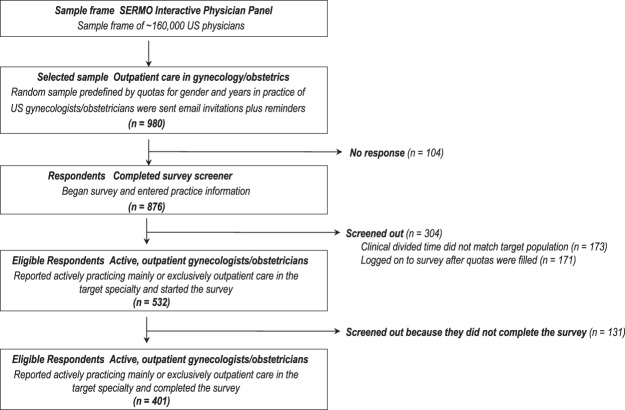
Respondent flow chart.

Of the 980 physicians invited, 104 did not respond and 876 started the survey. Of the 876 who started the survey, 475 were excluded: 173 indicated working mostly or exclusively in inpatient care, 171 logged on to the survey after the quota had been filled, and 131 did not complete the survey. That left 401 completed surveys for analysis (Fig. 3).

Using the AAPOR (American Association for Public Opinion Research) response rate calculator, which incorporates a default method for estimating the proportion of cases of unknown eligibility that is eligible (*e*) the survey yielded a response rate of 67.1% (401/[401 + 131 + *e*(104)]) and a cooperation rate of 75.4% (401/[401 + 131]). Gynecologists’ characteristics within the final sample matched the distribution of years in practice and gender of the AMA Masterfile (see Table 1).

### Survey Questionnaire, Intervention, and Outcome Measures

The survey first asked gynecologists if they regularly recommended ovarian cancer screening with TVU and CA-125 testing to asymptomatic, average-risk women. Gynecologists’ estimates and beliefs regarding screening’s benefit and harms were investigated by a series of questions that i) requested numerical estimates of the benefit (i.e., disease-specific mortality among every 1,000 with/without screening) and harms (e.g., percentage of false-positive test results out of all positive test results, overtreatment) of annual ovarian cancer screening over a period of 10 years, and ii) solicited their beliefs regarding the benefit-harm ratio (final version of the survey, see Supplementary Information). Estimates were rated as correctly approximating evidence if they were within a ± 10 percent margin of error of the figures reported for the PLCO trial^5^.

After responding to the questions, gynecologists were presented with a fact box summarizing the PLCO evidence on the effectiveness of ovarian cancer screening with ultrasound and CA-125 testing^5^ (see Fig. 2 in results). A fact box is a visual tabular display based on the PICO (Population, Intervention, Control, Outcome) model used in the context of evidence-based medicine and communicates the benefits and harms of medical interventions in absolute numbers adjusted to the same denominator to facilitate comprehension. Up to date, the format has been tested in laypeople only and shown to enhance even low-literate individuals’ understanding of medical facts^26,27,30^.

After taking as much time as desired to familiarize themselves with the contents of the fact box, gynecologists were asked whether seeing the evidence changed original estimations. If answering “yes,” they were asked the initial series of questions on the benefit and harms of screening again. If responding “no,” gynecologists exited the survey without being queried again.

The primary outcome measures were the proportion of gynecologists recommending screening, and the proportion of estimates (within a ± 10 percent margin of error) and beliefs regarding screening’s benefit and harms approximating current best evidence before and after intervention. The secondary outcome measures were the proportion of estimates/beliefs approximating current best evidence in dependence of gynecologists’ recommendation behavior and in dependence of their reaction to the evidence presented in the fact box, and the relationship between their reaction to the evidence presented in the fact box and their individual characteristics (years in practice, gender, and the proportion of initially provided correct estimations/beliefs out of all provided estimations/beliefs).

### Statistical Analysis

The questionnaire did not permit item nonresponse; all 401 questionnaires were complete. Results are provided as absolute frequencies and absolute proportions, respectively with 95% confidence intervals. Comparisons for estimates and beliefs derived before and after exposure to intervention were performed using the nonparametric Wilcoxon signed-rank test for dependent samples and the nonparametric McNemar test. Analyses of estimates and beliefs between gynecologists recommending/not recommending screening were performed using the nonparametric Pearson’s chi-square test and the nonparametric Mann-Whitney test. Logistic regression was used to investigate the relationship between gynecologists’ reaction to the evidence presented in the fact box and their individual characteristics (years in practice, gender, and the proportion of initially provided correct estimations/beliefs out of all provided estimations/beliefs). All data were stored and analyzed with IBM SPSS Statistics 24 (New York City, USA).

### Ethical approval

The study was approved by the Institutional Ethics Board of the Max Planck Institute for Human Development, Berlin (Germany).

## Electronic supplementary material

Supplementary Information

## Data Availability

The data set from which the results were derived can made available to authorized individuals upon written request to the authors.
