# Supplementary material for: US gynecologists’ estimates and beliefs regarding ovarian cancer screening’s effectiveness 5 years after release of the PLCO evidence
Source: Sci Rep. 2018 Nov 21;8:17181. doi: 10.1038/s41598-018-35585-z (PMC6249225; doi:10.1038/s41598-018-35585-z)
Supplement: Supplementary file 1 — Supplementary Information [file 41598_2018_35585_MOESM1_ESM.pdf]

US gynecologists' estimates and beliefs regarding ovarian cancer screening's effectiveness 5 years after release of the PLCO evidence

Odette Wegwarth*, PhD[a,b]; Gerd Gigerenzer, PhD[b]

[a]Max Planck Institute for Human Development, Center for Adaptive Rationality, Lentzeallee 94, 14195 Berlin, Germany, [b]Max Planck Institute for Human Development, Harding Center for Risk Literacy, Lentzeallee 94, 14195 Berlin, Germany

Supplementary Information

Table of contents

Survey………pp. 2 – 4

Additional Results: Prediction of Gynecologists' Reaction to Evidence Presented in the Facts Box Table by Their Characteristics (Years in Practice, Gender) and the Proportion of Initially Provided Correct Estimations/Beliefs…………………………………………………………………………………………..p. 5

Survey

Two qualifier questions:
1. What is your primary medical specialty?

Obstetrics & Gynecology (QUALIFIED)
Other specialty (NOT QUALIFIED)
Gynecology (QUALIFIED)

2. Which of the following best describes how your clinical time is divided?

() Exclusively outpatient (QUALIFIED)
() Mostly outpatient (QUALIFIED)
() Mostly inpatient (NOT QUALIFIED)
() Exclusively inpatient (NOT QUALIFIED)

Introduction

We know that you're busy and that you may get asked to complete a lot of surveys. But before deleting this, please take a minute to read on about why you might care about this particular survey.

What is this about? Through this survey, we hope to learn how physicians make decisions in the field of ovarian cancer screening and about the forces—including medical evidence—that influence their decisions.

Who is responsible? The survey is funded by Max Planck Institute for Human Development, Berlin (Germany) and conducted by GfK. The study has been approved by the Institutional Review Board of the Max Planck Institute for Human Development, Berlin.

What are we asking you to do? Please complete the enclosed survey, which should take about 20 minutes. As a token of our appreciation for completing this survey you will receive $50 for your participation in this important research.

Thank you very much.

[Start Survey]

[General recommendation]
QE1:
Do you regularly recommend ovarian cancer screening with transvaginal ultrasound and potentially with additional CA 125-testing to average-risk and asymptomatic women for early detection?

1. YES
2. NO

[Knowledge Benefit]
Imagine average-risk, asymptomatic women from the general public aged 55 to 75 years who attend or do NOT attend annual ovarian cancer screening with CA 125-testing and transvaginal ultrasound over a period of 10 years.

QWF1a:
How many of every 1,000 women NOT attending ovarian cancer screening regularly do you think will die of ovarian cancer within 10 years?
_____ out of 1,000 → QWF1b

QWF1b:
How many of every 1,000 women regularly attending ovarian cancer screening do you think will die of ovarian cancer within 10 years?
_____ out of 1,000 → QWF2a

[Knowledge Harms/ false alarms, overtreatment]
QWF2a:
Do you believe that ovarian cancer screening can harm a woman?

1. YES → QWF2b
2. NO → QWF3a

QWF2b:
What harm(s) could that be?
____(open-ended) →QWF3a

QWF3a:
How many of every 1,000 women regularly attending ovarian cancer screening over a period of 10 years do you think will receive a positive test result?
____ out of 1.000 → QWF3b

QWF3b:
How many of these positive test results do you think are false-positive test results?
___% of all positive test results are false-positive test results → QWF3c

QWF3c:
How many of these women who received a false-positive test result will have their ovaries removed as a consequence of further diagnostic work-up?
___% of all false-positive test results → QWF4

QWF4:
Do you think that the potential benefit of ovarian cancer screening (e.g., reduction of disease-specific mortality) outweighs the potential harms (e.g., false-positives, overdiagnosis)?

1. YES → QA1
2. NO → QA1

[INTERVENTION: FACT BOX] The following fact box summarizes data of the first and only randomized controlled trial on the effects of ovarian cancer screening with CA 125 testing and transvaginal ultrasound. The trial enrolled 78,216 women aged 55 to 74 years from the general U.S. population and had a mean follow-up of 13 years. For a period of 10 years, women in the screening group received CA 125 testing and transvaginal ultrasound, followed by standard care. Women in the nonscreening group received standard care only.

[Presentation of facts box, see Fig. 2 main document]

[Attendance to evidence]
QFB1b:
Has seeing the data presented in the fact box changed your original estimates of the benefit and harms of ovarian cancer screening?

1. YES → QFB1c
2. NO → Exit “Demography”

[Knowledge Benefit]
QFB1c:
How many of every 1,000 women NOT attending ovarian cancer screening regularly do you think will die of ovarian cancer within 10 years?

_____ out of 1,000 → QFB1d

QFB1d:
How many of every 1,000 women attending ovarian cancer screening regularly do you think will die of ovarian cancer within 10 years?
_____ out of 1,000 → QFB2a

[Harm/false positive, overtreatment]
QFB2a:
Do you think that ovarian cancer screening can also harm a woman?

1. YES → QFB2b
2. NO → QFB3a

QFB2b:
What harm(s) could that be?
____(open-ended) →QFB3a

QFB3a:
How many of every 1,000 women attending ovarian cancer screening over a period of 10 years do you think will receive a positive test result?
____ out of 1,000 → QFB3b

QFB3b:
How many of these positive test results do you think are false-positive test results?
___% of all positive test results are false-positive test results → QFB3c

QFB3c:
How many of these women who received a false-positive test result will have their ovaries removed as a consequence of further diagnostic work-up? → QFB4
___% of all false-positive test results

QFB4:
Finally, do you think that the potential benefit of ovarian cancer screening (e.g., reduction of disease-specific mortality) outweighs the potential harms (e.g., false-positives, overdiagnosis)?

1. YES
2. NO

Additional Results

Prediction of Gynecologists' Reaction to Evidence Presented in the Facts Box by Their Characteristics (Years in Practice, Gender) and the Proportion of Correct Estimations/Beliefs

To test whether gynecologists' changes in their initial estimations/beliefs after seeing the evidence in the facts box was associated with gynecologists' characteristics (years in practice, gender) and/or with the proportion of initially provided correct estimations/beliefs out of all of their provided estimations/beliefs, we used logistic regression. The individual proportion of correct estimations/beliefs was calculated by first coding "estimation of benefit," "estimation of false alarms," estimation of overtreatment," "belief of harms due to screening," and "expected benefit-harm ratio of screening" for each gynecologist as either 0 for "incorrect" or 1 for "correct" and then summing up these numbers on a scale of 0 to 5, where 0 indicates no correct response across the five questions and 5 indicates that all responses are correct across the five questions.

Analysis showed that the more years in practice gynecologists had spent and the fewer estimation/beliefs they had initially provided in accordance with current best evidence, the more likely they were to change their estimations/beliefs after facts box presentation (**see Regression Table**).

		***95% CI* for Odds Ratio**			
	***B* (SE)**	**Lower**	**Odds Ratio**	**Upper**	**Sig.**
**Change vs. No Change of Initial Estimates and Beliefs After Seeing Evidence**					
Intercept	- 0.12 (0.35)				.728
Proportion of Correct Estimations/Beliefs	0.46 (0.13)	1.24	1.59	2.03	< .001
Years in Practice	- 0.27 (0.10)	0.63	0.76	0.92	< .01
Gender	- 0.02 (0.21)	0.61	0.98	1.48	.923
Proportion of Correct Estimations/Beliefs* Years in Practice	0.07 (0.12)	0.85	1.08	1.36	.536

Note: R^2 = .06 (Cox & Snell), 0.08 (Nagelkerke), Model χ^2 (4) = 24.94, p = <.001.
